# Mental health and wellbeing outcomes associated with social, medical, and legal gender affirmation among trans young people in Australia

**DOI:** 10.1080/26895269.2024.2366881

**Published:** 2024-06-19

**Authors:** Ruby Grant, Natalie Amos, Ashleigh Lin, Teddy Cook, Adam O. Hill, Ken Pang, Rachel S. Skinner, Marina Carman, Adam Bourne

**Affiliations:** a Australian Research Centre in Sex, Health, and Society, La Trobe University, Melbourne, Australia; b School of Population and Global Health, University of Western Australia, Perth, Australia; c ACON Health, Surry Hills, Australia; d Graduate School of Public Health, St Luke’s International University, Tokyo, Japan; e Population Health and Clinical Sciences Themes, Murdoch Children’s Research Institute, Parkville, Australia; f Department of Paediatrics, University of Melbourne, Parkville, Australia; g Royal Children’s Hospital Department of Adolescent Medicine, Parkville, Australia; h School of Medicine, Specialty of Child and Adolescent Health, University of Sydney, Sydney, Australia; i The Kirby Institute, UNSW Sydney, Sydney, Australia

**Keywords:** Gender affirmation, hormone therapy, legal gender recognition, transgender, youth

## Abstract

**Background:**

Gender affirmation is known to improve mental health among trans adults, but its impact on young people has been questioned. This study examined the mental health outcomes associated with social, medical, and legal gender affirmation among trans young people in Australia.

**Methods:**

Data from 1697 trans young people aged 14–21 who participated in an Australian national survey were analyzed using multivariable logistic regression analyses. We measured associations between gender affirmation and various mental health variables, including levels of happiness, psychological distress, anxiety, self-harm and recent suicidal ideation and suicide attempts. Associations between gender affirmation and young people’s wellbeing, including experiences of verbal harassment, homelessness, and drug use were also examined.

**Results:**

Participants who had affirmed their gender legally and medically (*via* hormone therapy or surgery/ies) reported lower levels of anxiety and psychological distress, while legal gender affirmation was associated with lower suicidal ideation. Social affirmation was not directly associated with wellbeing outcomes but was linked to an increased likelihood of verbal harassment and homelessness. All forms of gender affirmation were associated with increased drug use, which likely reflects the greater opportunities for social connection and LGBTQ-community involvement that trans young people experience when they take steps to affirm their gender.

**Conclusion:**

All forms of gender affirmation were positively associated with the wellbeing of trans young people. Though young people who had socially affirmed their gender faced higher likelihoods of experiencing verbal harassment and homelessness, this is likely linked to their increased visibility and subsequent vulnerability to discrimination. These findings highlight the need for targeted interventions to address transphobia and discrimination. A comprehensive approach to supporting the wellbeing of trans young people should include legal, medical, and social affirmation, as well as addressing potential negative outcomes *via* societal education.

## Introduction

Trans people of all genders may choose to pursue a range of pathways to affirm their gender at various points throughout their lives. Common approaches to affirmation include social (e.g. choosing names and pronouns that reflect your gender, and wearing clothes, hairstyles, and making other cosmetic changes to express your gender), medical (e.g. puberty blockers, hormone therapies, and surgeries), and legal (e.g. amending your name and/or sex/gender on official identity documents such as your birth certificate or passport). Research has consistently shown that gender affirmation promotes positive mental health outcomes for trans people of any age (Olson-Kennedy et al., 2018; Russell et al., 2018). Notably, the benefits of medical gender affirmation are particularly well-documented for adults (Gorin-Lazard et al., 2012; Mahfouda et al., 2019), and there is also a growing body of work indicating similar benefits for young people (Chen et al., 2023; De Vries et al., 2014). These benefits also extend to social (Brown et al., 2020) and legal affirmation (Fontanari et al., 2020), though these areas have received comparatively less scholarly attention. Examining the mental health outcomes associated with different forms of gender affirmation for young people is necessary to confirm the value of these practices and support the validity ongoing access to gender affirming care, particularly for young people who often experience age-specific barriers to affirmation depending on jurisdiction (Riggs et al., 2020). Therefore, this article examines the mental health and wellbeing outcomes associated with different forms of gender affirmation (social, medical, legal) for young people, using Australia’s largest current dataset of trans youth (aged 14–21).

Many trans people may begin questioning their gender in childhood and adolescence (Turban et al., 2023), during which time they can experience feelings of acute physical and emotional distress related to the incongruence between their gender identity and sex presumed at birth (Atkinson & Russell, 2015). Such negative feelings may also be exacerbated by exposure to stigma against trans people, which can take the form of casual negative remarks, negative media representations, explicit and implicit discrimination in a range of settings, and experiences of harassment or abuse (Hughto et al., 2015). Anti-trans stigma may also contribute to common instances of family rejection that many trans young people experience (Klein & Golub, 2016). This arguably results in higher rates of homelessness for this population (Lim et al., 2023), and exacerbates a range of poor mental health outcomes including increased anxiety, psychological distress, self-harm, and suicidality (Adams et al., 2017; Hill et al., 2023a; Strauss et al., 2020). In line with the notion of minority stress (Meyer, 2003), previous research also reflects increased drug use among this population, often as a form of coping with the compounding effects of the stressors outlined above (Bretherton et al., 2021). Nevertheless, trans communities and their allies have identified and established a range of support mechanisms to address stigma and promote greater wellbeing for this group.

Trans affirming practices across a range of social and institutional contexts have been shown to improve trans young people’s mental health and wellbeing. For example, qualitative studies indicate that social support, including use of gender affirming language, from family, friends, and in school environments, is linked to reduced suicide risk in trans young people (Carlile et al., 2021; Fuller & Riggs, 2018). Access to gonadotropin-releasing hormone analogues, or, puberty blockers, for trans adolescents has been linked to improved wellbeing (Rew et al., 2021; Riggs et al., 2020). Similarly, both hormone therapies and gender affirming surgical procedures have been shown to increase life satisfaction while reducing anxiety and depression among trans young adults (Chen et al., 2023; Mahfouda et al., 2019). Legal gender affirmation can help to validate trans people’s identities, removing barriers to accessing necessary healthcare and other services (Lelutiu-Weinberger et al., 2020; Scheim et al., 2020). For example, Puckett et al. (2024) US survey of trans people (aged 19+) found that those who had legally affirmed their gender reported less enacted stigma, resulting in lower rates of psychological distress and physical health issues. Fontanari et al. (2020) survey of 350 Brazilian trans youth found that those who had legally changed their name were 31% less likely to be experiencing anxiety and 13.5% less likely to have a mood disorder compared to those who had not changed they name. However, quantitative research on the mental health benefits associated with legal affirmation, particularly for young people, remains limited. Further research is therefore necessary to examine the mental health and wellbeing outcomes associated with different forms of gender affirmation for trans young people in Australia.

Trans people in Australia are afforded a range of supports, rights and protections at state and national level. State-based LGBTQA-inclusion policies and government strategies are increasingly common across all eight states and territories. Australia’s universal public healthcare system (Medicare) allows for affordable or free general medical care and subsidized prescription medications for all citizens and permanent residents, including access to hormone replacement therapies. However, access to medical gender affirmation for people under the age of 18 previously required authorization by the Family Law Court, though this is now accessible at the discretion of treating clinicians with parental consent, making access without parental support difficult (Riggs et al., 2020). Medical gender affirmation for young people is available in some states through specific gender clinics or services within major children’s hospitals, or through general practitioners in other areas. Requirements for legal gender affirmation vary by jurisdiction, with changes to birth certificates and drivers’ licenses managed by states and changes to passports managed federally. Each state has different requirements for legal affirmation, with some states only allowing changes to birth certificates once an individual has received gender affirming genital surgeries. While it varies from state to state, most states require young people to be at least 16 years old, or have consent from both parents, before they can legally affirm their gender. With this context in mind, this article will explore the retrospective social, medical, and legal gender affirmation experiences of trans young people (aged 14-21) and their current wellbeing outcomes.

## Methods

### Sample and procedure

The study sample was drawn from *Writing Themselves In 4*, a national survey of 6418 lesbian, gay, bisexual, trans, queer and asexual (LGBTQA) youth aged 14–21 in Australia. The present paper analyses the data of 1697 trans participants who answered the section of the survey specific to trans experiences. The survey was developed in close consultation with a Community Advisory Board and Youth Advisory Group, including representation of trans young people and community organizations. The survey was completed online, open to responses in late 2019 and promoted *via* targeted social media advertising as well through LGBTQA community organizations. Beyond eligibility criteria, all survey questions were optional. Ethics approval was obtained from the La Trobe University Human Research Ethics Committee.

### Measures

#### Demographic factors

Demographic information collected in the study included age, gender, sexual orientation, current level of education and location of residence.

Sexual orientation was evaluated by asking participants to select the term that best aligned with their sexual orientation from 12 available options: ‘gay’, ‘lesbian’, ‘bisexual’, ‘pansexual’, ‘queer’, ‘asexual’, ‘homosexual’, ‘heterosexual’, ‘prefer not to answer’, ‘prefer not to have a label’, ‘don’t know’, and ‘something different.’ Due to small sample sizes, those who identified as homosexual or preferred not to have a label were consolidated into the ‘something different’ category.

To determine gender identity, participants were asked to select from a list of 17 gender descriptors that best represented them. Gender modality (whether participants were cisgender or trans) was then established based on the responses from participants about the gender on their original birth certificate and their answers to the gender identity question. Gender categories were cisgender woman (participants assigned female at birth and who identified solely as ‘woman’), cisgender man (participants assigned male at birth and who identified solely as ‘man’), trans woman (participants assigned male at birth and who identified solely as ‘woman’, ‘trans woman’, or ‘sistergirl’), trans man (participants assigned female at birth and who identified solely as ‘man’, ‘trans man’, or ‘brotherboy’), and non-binary (participants who identified with a gender that wasn’t binary or expressed that they could not choose a singular gender identity). These categories were established following extensive consultation to ensure they sensitively reflected understandings of trans communities nationally. However, we note that perspectives on these definitions may vary within communities. For instance, our inclusion of Australian Indigenous trans identities sistergirl and brotherboy within the respective Western analytic categories of trans women and trans men may not reflect all communities’ understandings of those identities (Riggs & Toone, 2017). Nevertheless, these were categorized thus given their smaller sample sizes and in consultation with trans Aboriginal and Torres Strait Islander community members.

#### Gender affirmation

Trans participants were asked if they had ever wanted to affirm their gender in the following ways, with options to choose from ‘Socially (i.e. change your name/pronouns or gender presentation’; ‘Legally (i.e. change your legal name or gender markers on ID documents)’; ‘Medically (i.e. puberty blockers, hormone therapy, gender affirming surgeries)’ or ‘None of the above.’ Participants were able to select more than one form of gender affirmation in response to this question. Those who had desired to affirm their gender in any of these ways were further asked if they had ever done so.

#### Suicidal ideation and attempt

To evaluate experiences related to suicidal ideation and attempts, participants were queried if they had had any thoughts of suicide or a desire to end their lives, and whether they had made any suicide attempts or efforts to end their lives. Available responses encompassed ‘No,’ ‘Yes, in the past 12 months,’ ‘Yes, more than 12 months ago,’ and ‘Prefer not to answer.’ Participants were allowed to choose multiple responses, unless they selected ‘No’ or ‘Prefer not to answer.’ To focus on recent instances of suicidal ideation and attempts, binary variables were created that indicated whether participants had experienced such thoughts or attempts in the past 12 months.

#### Self-harm

To examine experiences related to self-harm, participants were asked whether they had experienced thoughts about harming themselves on purpose, and whether they had injured or harmed themselves on purpose. Available responses included ‘No, never,’ ‘Yes, in the past 12 months,’ ‘Yes, more than 12 months ago,’ and ‘Prefer not to answer.’ Participants were able to choose multiple responses, unless they selected ‘No’ or ‘Prefer not to answer.’ To focus on recent instances of self-harming, a binary variable was created to indicate whether participants had injured or harmed themselves in the past 12 months.

#### Psychological distress

The 10-item Kessler Psychological Distress Scale (K10) (Kessler et al., 2002) was employed to gauge current levels of psychological distress. The K10 scale investigates symptoms of anxiety or depression that participants might have encountered in the preceding four weeks. Participants were requested to rate their experiences using a 5-point Likert scale, spanning from ‘None of the time’ to ‘All of the time.’ The overall scores on the scale can vary from 10 to 50, with heightened scores signifying more intense psychological distress. For the purposes of our analysis, this was used as a continuous variable. At the time of writing, the K10 has yet to be validated for use with LGBTQA populations, and normative data for LGBTQA populations is unavailable. However, the K10 is generally considered valid and appropriate for use with these populations (Tan et al., 2022)

#### Anxiety

Generalized anxiety was assessed using the Generalized Anxiety Disorder Assessment (GAD-7) (Spitzer et al., 2006). The GAD-7 is a seven-item scale which asks respondents to indicate the frequency at which they have experienced symptoms of anxiety. Scores can range from 0 to 21, with 0 indicating no anxiety and higher scores indicating higher levels of anxiety. The GAD-7 is commonly used to examine experiences of anxiety among LGBTQ people (Bavinton et al., 2022; Borgogna et al., 2019; Flentje et al., 2020). For the purposes of our analysis, this was used as a continuous variable.

#### Subjective happiness

Subjective happiness was gauged using the 4-item self-report, globally validated Subjective Happiness Scale (SHS) (Shimai et al., 2004). This scale has been widely used in previous studies of LGBTQA populations (Greene & Britton, 2015; Strizzi et al., 2016). Participants were asked to respond to items on a 7-point Likert scale, such as “In general I consider myself…” with potential answers varying from 1 “Not a very happy person” to 7 “A very happy person.” Following the reverse coding of a negatively worded item, an average was calculated from the responses to all 4 items. The scores ranged from 1 to 7, with a higher score suggesting increased happiness (Lyubomirsky & Lepper, 1999). For the purposes of our analysis, this was used as a continuous variable.

#### Drug use

Participants were asked if they had used any drugs other than alcohol or tobacco in the past six months for non-medicinal purposes (i.e. not prescribed them by a doctor). A six-month time frame was used due to literature observing shorter time frames having more reliable recall about drug use (Janssen et al., 2017). Participants selected all that applied from a list of drugs, including cannabis, cocaine, ecstasy/MDMA, antidepressants, benzodiazepines, GHB, ketamine, amyl nitrate, antipsychotics, pharmaceutical opioids, steroids, hallucinogens, LSD, meth, heroin, nitrous oxide, and mephedrone. Participants also had the option to additionally indicate other drugs not listed or no drug use. Responses to this question were coded as no drug use or any drug use in the past six months.

#### Homelessness

To evaluate instances of homelessness, participants were questioned if they had ever: fled from home or their residence; vacated home or their dwelling because they were requested or compelled to leave; resorted to couch surfing due to the lack of an alternative place to stay; or experienced homelessness. Borrowing from a previous study of youth homelessness in the United States (Perlman et al., 2014), homelessness was defined to participants as ‘not having a stable or safe place to live and can include things like sleeping outside, and living or sleeping in a car, shelter, hostel, or refuge’. Participants who responded ‘yes’ to any of the previous situations were then asked whether they were currently facing this situation, if it had happened within the past 12 months, or if it was more than 12 months ago, respective to each response. For the context of the current study, the responses were classified as having experienced homelessness at any point or not.

#### Experiences of verbal harassment in the past 12 months

Participants were asked if they had been harassed or assaulted in the past 12 months based on their sexuality or gender identity, and provided with a list of several forms of harassment including verbal abuse (such as being called names or threatened). Participants were asked to indicate whether or not they had experienced these forms of harassment in the past 12 months.

### Statistical analysis

Statistical evaluations were performed utilizing Stata (Version 16.1 SE; StataCorp, College Station, TX). Descriptive statistics provided an overview of the sample’s sociodemographic attributes.

First, Chi-square analyses were performed to establish if there were difference in the rates of desire for and access to medical, legal, and social gender affirmation across genders (trans men, trans women and non-binary). To identify specific differences between each pair of gender groups, pairwise comparisons were performed post hoc. A Bonferroni correction was applied to adjust for multiple comparisons, with significance determined at *p* < 0.0167. Next a series of multivariable linear and logistic regression analyses were used to explore the mental health and wellbeing outcomes of trans and gender diverse youth who had affirmed their gender medically, legally or socially, among those who had desired to affirm their gender in these ways. These analyses allowed us to examine the relationship between the predictor variables (access to medical, legal or social gender affirmation) and the outcome variables, while controlling for the potential impact of confounding factors. Regression analyses were run individually for each form of gender affirmation (medical, legal or social). Linear regression analyses were used when the outcome variables were continuous, these included level of psychological distress, anxiety scores and subjective happiness scores. Logistic regression analyses were used when the outcome variables were dichotomous, these included past 12-month suicidal ideation, past 12-month suicide attempt, past 12-month self-harm, past six-month drug use, ever experiencing homelessness and past 12-month experiences of verbal harassment. Each of the regression models controlled for the potential confounding effects of demographic traits including age, gender, sexual orientation, level of education and residential location. In addition, all regression analyses except for those with verbal harassment as the outcome, controlled for the potential confounding effects of past-12 month verbal harassment.

## Results

Table 1 presents the demographic characteristics of the sample, which is diverse across a number of traits including age, gender, sexual orientation, level of education and residential location.

**Table 1.** Sample characteristics (*n* = 1697).

	*n*	%
Age group		
14-17	945	55.7
18-21	752	44.3
Sexual orientation		
Lesbian	148	8.7
Gay	151	8.9
Bisexual	365	21.5
Pansexual	332	19.6
Queer	261	15.4
Asexual	113	6.7
Something else	326	19.2
Gender		
Trans woman	75	4.4
Trans man	406	23.9
Non-binary	1216	71.7
Education level		
Secondary school (high school)	929	58.9
University	375	23.8
TAFE	151	9.6
Other	121	7.7
Country of birth		
Australia	1535	90.6
Other English-speaking country	97	5.7
Non-English-speaking country	62	3.7
Residential location		
Capital city, inner suburban	99	5.8
Capital city, outer suburban	916	54.1
Regional city or town	474	28
Rural/remote	205	12.1

Table 2 provides a breakdown of the frequencies of hoping to and actively affirming gender medically, legally, or socially across gender identities (trans men, trans women and non-binary). Chi-square analyses revealed significant differences in desire for and access to each form of affirmation between genders with non-binary people reporting considerably lower desire for and access to medical and legal affirmation than trans men and women, as well as a lower frequency of having socially affirmed.

**Table 2.** : Frequencies of hoping to and actively affirming gender medically, legally, or socially.

	Trans woman		Trans man		Non-binary		Total		
	*n*	%	*n*	%	*n*	%	*n*	%	Χ^2^
Medical affirmation									
Hoped to affirm	71	98.6	393	98.0	558	59.5** ^a^	1022	72.4	234.7**
Actively affirmed	34	47.9	177	44.1	90	9.7** ^b^	301	21.5	227.0**
Legal affirmation									
Hoped to affirm	68	94.4	394	98.3	602	64.2** ^c^	1064	75.4	190.7**
Actively affirmed	19	26.8	132	32.9	89	9.6** ^d^	240	17.2	111.7**
Social affirmation									
Hoped to affirm	71	98.6	401	100.0	904	96.4** ^e^	1376	97.5	15.6**
Actively affirmed	53	74.6	369	92.0** ^f^	609	65.8** ^g^	1031	73.7	99.6**

** <0.001;

^a^differs to trans women (Χ^2^ = 43.6) and trans men (Χ^2^ = 202.5);

^b^differs to trans women (Χ^2^ = 88.2) and trans men (Χ^2^ = 206.3);

^c^differs to trans women (Χ^2^ = 27.4) and trans men (Χ^2^ = 171.2);

^d^differs to trans women (Χ^2^ = 20.08) and trans men (Χ^2^ = 109.5);

^e^differs to trans men (Χ^2^ = 14.9);

^f^differs to trans women (Χ^2^ = 19.2);

^g^differs to trans men (Χ^2^ = 99.5);

Table 3 presents the multivariable logistic regression and linear regression results exploring associations between access to medical, legal, and social gender affirmation and mental health and wellbeing outcomes**.**

**Table 3.** Associations between access to medical, legal or social gender affirmation and health and wellbeing outcomes.

	Recent suicidal ideation				Recent suicide attempt				Recent self-harm			
	*n*	%	AOR(95% CI)	*p*	*n*	%	AOR(95% CI)	*p*	*n*	%	AOR(95% CI)	*p*
Affirmed medically												
No	791	75.0	REF	–	145	14.4	–	–	592	54.8	–	–
Yes	212	72.6	0.8 (0.54 − 1.19)	0.275	50	17.8	1.06 (0.64 − 1.74)	0.821	154	51.3	0.89 (0.62 − 1.28)	0.529
Affirmed legally												
No	842	75.4	REF	–	157	14.7	–	–	628	55.0	–	–
Yes	161	70.0	0.59 (0.39 − 0.89)	0.012	38	17.3	0.99 (0.59 − 1.66)	0.980	118	49.4	0.79 (0.54 − 1.14)	0.204
Affirmed socially												
No	263	74.1	REF	–	41	12.2	–	–	187	51.7	–	–
Yes	740	74.6	0.79 (0.55 − 1.13)	0.198	154	16.2	1.15 (0.71 − 1.87)	0.558	559	54.9	1.07 (0.79 − 1.44)	0.665
	Psychological distress				Anxiety				Happiness			
			β(95% CI)	*p*			β(95% CI)	*p*			β(95% CI)	P
Affirmed medically												
No	–	–	REF	–	–	–	–	–	–	–	–	–
Yes	–	–	−1.63 (−2.99 – −0.27)	0.019	–	–	−1.01 (−1.96 – −0.06)	0.038	–	–	0.03 (−0.21 − 0.28)	0.805
Affirmed legally												
No	–	–	REF	–	–	–	–	–	–	–	–	–
Yes	–	–	−2.68 (−4.05 – −1.31)	0.000	–	–	−1.39 (−2.37– −0.4)	0.006	–	–	0.32 (0.07 − 0.56)	0.011
Affirmed socially												
No	–	–	REF	–	–	–	–	–	–	–	–	–
Yes	–	–	−1.05 (−2.22 − 0.11)	0.077	–	–	−0.43 (−1.23 − 0.37)	0.289	–	–	0.17 (−0.03 − 0.36)	0.090
	Drug use						Homelessness				Verbal harassment	
	n	%	AOR(95% CI)	*p*	*n*	%	AOR(95% CI)	*p*	*n*	%	AOR(95% CI)	*p*
Affirmed medically												
No	301	30.2	REF	–	349	32.1	–	–	607	57.0	–	–
Yes	128	46.9	1.75 (1.17 − 2.6)	0.006	120	40.4	1.04 (0.7 − 1.53)	0.856	177	60.4	1.22 (0.82 − 1.83)	0.324
Affirmed legally												
No	330	31.5	REF	–	383	33.3	–	–	645	57.3	–	–
Yes	99	44.6	1.57 (1.06 − 2.33)	0.025	86	36.8	0.91 (0.61 − 1.36)	0.653	139	59.7	1.21 (0.8 − 1.82)	0.368
Affirmed socially												
No	87	26.3	REF	–	85	23.4	–	–	187	53.1	–	–
Yes	342	36.4	1.52 (1.04 − 2.21)	0.030	384	37.6	1.53 (1.06 − 2.21)	0.022	597	59.3	1.85 (1.37 − 2.5)	0.000

**NOTE:** n’s and %’s reflect the frequency and proportion of participants who had experienced the outcomes. This only applies to dichotomized outcomes used in the logistic regression analyses.

### Mental health and wellbeing

Trans young people who had undergone medical (β = −1.63, CI = −2.99–0.27, *p* = 0.019) and legal (β = −2.68, CI = −4.05–1.31, *p* < 0.001) affirmation of their gender had lower K10 psychological distress scores, but this was not the case for those who affirmed their gender socially. Additionally, young people who had affirmed their gender medically (β = −1.01, CI = −1.96–0.06, *p* = 0.038) and legally (β = −1.39, CI = −2.37–0.40, *p* = 0.006) had lower anxiety scores on the GAD-7, but not those who affirmed their gender socially. We also found that young people who legally affirmed their gender had higher happiness scores on the SHS than those who had not (β = 0.32, CI = 0.07-0.56, *p* = 0.011), but there were no associations between medical or social affirmation and SHS scores.

### Suicidality and self-harm

Participants who had legally affirmed their gender experienced a reduced likelihood of having suicidal ideation in the past year (AOR = 0.59, CI = 0.39–0.89, *p* = 0.012). However, there were no significant associations observed between medical and social affirmation and recent suicidal ideation. Furthermore, none of these forms of gender affirmation were significantly associated with suicide attempts or self-harm.

### Drug use

All forms of gender affirmation were associated with a higher likelihood of drug use: medical affirmation (AOR = 1.75, CI = 1.17–2.60, *p* = 0.006); legal affirmation (AOR = 1.57, CI = 1.06–2.33, *p* = 0.025); and social affirmation (AOR = 1.52, CI = 1.04–2.21, *p* = 0.030).

### Homelessness

Participants who had socially affirmed their gender were more likely to have experienced homelessness (AOR = 1.53, CI = 1.06–2.21, *p* = 0.022), but this was not the case for those who had affirmed their gender medically or legally.

### Verbal harassment

Young people who had socially affirmed their gender were more likely to have experienced verbal harassment based on their gender or sexual identity in the past 12 months (AOR = 1.85, CI = 1.37–2.5, *p* < 0.001). However, this was not the case for those who had affirmed their gender medically or legally.

## Discussion

Gender affirmation was a common goal for our participants, with the vast majority hoping to affirm their gender socially (97.5%), medically (72.4%), and legally (75.4%). However, in all cases, the frequency of those actively affirming their gender was much lower than those hoping to do so. Fewer participants had accessed legal affirmation (17.2%), while social affirmation was common (73.7%). This likely reflects the fact that currently in Australia access to legal affirmation for those under the age of 18 can be challenging without significant familial support. As most states only allow legal gender affirmation once medical gender affirmation (and in some cases, only surgical affirmation) has commenced, this option is much less accessible for many trans young people. Previous research in the US found that trans women who were able to access legal affirmation had higher average incomes and greater housing stability, reflecting an increased level of economic capital and social support likely required to access this form of affirmation (Hill et al., 2018). This, along with the benefits of gender affirmation that we and others document (Riggs et al., 2020), reflects the need for increased support for young people to access legal and medical gender affirmation in a timely manner. Information and guidance are also necessary for parents and guardians to support trans young people in their care to access different forms of gender affirmation should they wish.

Among our participants, trans men were the most likely to have socially and legally affirmed their gender, while trans women were the most likely to have medically affirmed their gender. Although a similar amount of non-binary young people hoped to socially affirm their gender as trans men and trans women, they were less likely to have actively done so. Non-binary participants were also much less likely to seek and achieve medical or legal affirmation. While these differences likely reflect variations in non-binary young people’s experiences of gender, they may also indicate access barriers to gender affirmation for non-binary people (Kennis et al., 2022). For example, previous research points to a level of medical gatekeeping on the part of some healthcare providers who may have preconceived notions about what an ‘ideal’ gender affirmation journey looks like (Grant et al., 2023). Such notions might focus on ‘transnormative’ or binary gender ‘transitions’ rather than more fluid gender affirmation approaches (Latham, 2019). An additional barrier non-binary people face in Australia is that some states do not allow legal gender affirmation for non-binary people, without having undergone genital surgeries, which some non-binary people do not seek (Kennis et al., 2022). Research examining non-binary young people’s specific gender affirmation experiences and needs remains sparce (for an exception, see Chew et al., 2020), with our findings suggesting the need for greater consideration of non-binary young people in research, policy, and healthcare provision.

Our findings extend existing understandings of trans young people’s experiences of gender affirmation by analyzing the mental health and wellbeing outcomes associated with different types of affirmation (social, medical, and legal). Notably, we found that legally affirming one’s gender was most strongly associated with greater wellbeing for trans young people, correlating with low rates of anxiety, psychological distress, and suicidal ideation, as well as greater overall happiness (see also Fontanari et al., 2020; Puckett et al., 2024). Given the level of familial support required to access legal gender affirmation in Australia as a young person, it is likely that this is another factor contributing to the overall increased wellbeing that we see associated with legal affirmation. In line with past research (Green et al., 2022), access to medical gender affirmation (i.e. puberty blockers, hormones, and/or surgeries), was also linked to lower anxiety and distress. In contrast with Fontanari et al. (2020), and the findings of past qualitative work (Brown et al., 2020), we found that social affirmation alone was not always strongly associated with improved wellbeing. In fact, young people who had socially affirmed their gender were more likely to experience verbal harassment and homelessness than those who had not affirmed their gender. This may be due to the timing of affirmation. Social affirmation may coincide with when a young person first discloses their gender to their family and friends (Haimson & Veinot, 2020). This period is when they are likely to be at greatest risk of family abandonment leading to homelessness (Lim et al., 2023). At the same time, in the absence of medical affirmation, a young person’s physical appearance may be less congruent with their gender identity and expression, making them greater targets for harassment (Grossman et al., 2009; McGuire et al., 2010). This confirms the ongoing need to promote awareness of trans affirming practices in social life more broadly, ensuring that trans young people are treated with dignity and respect regardless of how they affirm their gender.

In addition to mental health outcomes, we also analyzed associations between gender affirmation and drug use as a wellbeing variable. Previous research has drawn varied associations between mental ill-health and substance use in both the general population (Esmaeelzadeh et al., 2018) and among LGBTQA people collectively (Hill et al., 2023b; Roxburgh et al., 2016). Trans adults, in particular, report higher rates of drug use compared to cisgender heterosexual and cisgender LGBQA people (Connolly & Gilchrist, 2020). Some studies of trans young people’s drug use also reflect patterns of higher drug use compared with their cisgender heterosexual peers, though not necessarily higher than cisgender LGBQA young people (Fahey et al., 2023). Within trans communities, Turban et al. (2022) found that trans adults who had medically affirmed their gender had higher rates of alcohol and drug use than those who had not. Similarly, we also found that all forms of gender affirmation were associated with increased drug use among the young people in our sample. Previous research has often deployed the notion of minority stress (Lea et al., 2014; Meyer, 2003) as an explanation for this higher drug use among trans populations, with drug use providing a coping mechanism for those experiencing discrimination or marginalization based on their gender and/or sexuality (Connolly & Gilchrist, 2020; Fahey et al., 2023). However, given that medical and legal affirmation were associated with reduced distress and anxiety in our sample, if drug use was entirely due to minority stress, we might expect it to decline, not increase, as participants affirmed their gender in these ways. A possible alternative explanation is that trans young people who take steps to affirm their gender may be more socially connected (Cardona et al., 2023), thus having greater ability to participate in rites-of-passage common among young people, including experimenting with alcohol and other drugs (Kidd et al., 2018). While trans people who have not affirmed their gender report high rates of social isolation (Bowling et al., 2020), those who have affirmed their gender may be more connected with LGBTQA community groups where substance use can be normalized (Fahey et al., 2023). Following this interpretation, trans young people’s drug use must be understood as a cultural practice that may provide meaningful social connections for this often-marginalised group (Roxburgh et al., 2016). The role of drug use in trans young people’s experiences of gender affirmation requires further investigation, and services supporting trans young people must acknowledge young people’s own understandings of drug use. Both mainstream and LGBTQA-specific drug and alcohol support services must be trans affirming and open to providing tailored supports that respond to the varied roles that drug use may play in trans young people’s lives and gender affirmation journeys.

### Limitations

This study provides essential new insights into trans young people’s experiences by measuring the mental health and wellbeing outcomes associated with different types of gender affirmation. Few studies have explored the mental health and wellbeing outcomes associated with legal gender affirmation for trans young people. Despite its important contributions, this work is not without limitations. For instance, the convenience sampling methodology used means that our results are not representative of all trans young people in Australia, possibly excluding those who are not yet out, those who may not have felt safe to complete a survey about LGBTQA experiences, or who are not connected with LGBTQA communities or groups. In particular, our trans sample included a high representation of non-binary young people, a group with distinct gender affirmation preferences and experiences (Kennis et al., 2022) that likely skew our results. Additionally, despite extensive community consultation and targeted recruitment efforts, trans women make up a very small portion of the overall sample. Careful interpretation is thus required when generalizing these findings to the broader trans population. Furthermore, because our sample includes those aged 14–21 and we did not ask when participants accessed different forms of gender affirmation, we are not able to differentiate between those who had affirmed their gender as minors and those who had affirmed their gender as young adults. Notably, this study was cross-sectional and we are therefore unable to ascertain the direction of relationships between mental health outcomes and gender affirmation. Nonetheless, the study draws from the largest survey sample of trans young people in Australia, which is able to provide meaningful insights into the importance of gender affirmation for this population.

## Conclusion

Our results confirm that overall, gender affirmation is linked to positive health and wellbeing outcomes for trans young people. Trans young people may face challenges when seeking to socially affirm their gender, highlighting the importance of promoting trans affirming practices in the general community. However, those who access medical and legal affirmation reported improved wellbeing. Correlations between all forms of gender affirmation and drug use likely reflect trans young people’s greater ability to socialize and participate in common rites-of-passage once they have affirmed their gender. Nevertheless, further research is required to examine trans young people’s understandings of drug use within the context of their gender affirmation. Such information is necessary to inform drug education and support services that are trans-inclusive as well as ensuring gender affirming care that sensitively responds to trans young people’s drug use. Increasing access to social support for trans young people, providing information for families, and expanding medical and legal affirmation options for young people is essential for the wellbeing of this population.
